# Relationship between Attachment Styles and Risk Behavior (Smoking and Bullying) among Secondary School Students: An Experience from Hulu Langat, Malaysia

**DOI:** 10.18502/ijph.v49i10.4708

**Published:** 2020-10

**Authors:** Marjan MOHAMMADZADEH, Hamidin AWANG, Cheah Yt JUN, Nor Fauziah HASHIM, Archana PREMKUMAR

**Affiliations:** 1. Department of Community Health, Faculty of Medicine and Health sciences, Universiti Putra Malaysia, Malaysia; 2. Department of Psychiatry, Faculty of Medicine and Health sciences, Universiti Putra Malaysia, Malaysia

## Dear Editor-in-Chief

Attachment theory highlights the needs of humans in neonatal period, in order to interpret the specific protection and security seeking behaviors in this period of human life (1).On the other hand, adolescence indicates a challenging bridge between childhood and adulthood along with several biological, behavioral, emotional, and cognitive changes and challenges (2–3).In adolescence, the patterns of attachment-related behaviours are changed because the nature of individuals’ relations mostly shift from parental-relationship focused to peer-relationship focused. This is a normal developmental step coming with the improving of cognitive maturity (4). Attachment style are generally are classified as 2 main patterns including secure (for example an infant felt safe and under support with parents) and insecure attachment styles including “Anxious-resistant insecure attachment style” (also is called ambivalent attachment) and “Anxious-avoidant insecure attachment”. Anxious-avoidant insecure attachment style is represented an infant/child avoiding or showing little emotions to parents or caregivers (for example when they depart or return home) while an infant/child with anxious-resistant attachment style is mostly wary of strangers, the child is often highly distressed when caregivers depart but ambivalence when they return (5). Previous studies have approved the effects of parents-adolescent attachment style on adolescents’ psychosocial and behavioral development (6–7). However, there is still lack of information about the influence of attachment style on different factors of adolescents’ risk behavior (8).

The current cross-sectional study was amid to investigate the associations between three adolescents’ risk behaviors including smoking and bullying. Overall, 1800 male and female Malaysian adolescents (aged 13 to 17 yr old), from 10 randomly-selected secondary school in Hulu Langat, Malaysia (2012–2013) participated in the study. The validated Malay version of self-administered Inventory of Parent and Peer Attachment (IPPA) and Youth Risk Behaviors Surveillance Questionnaire (focusing on smoking and bullying) were used as the study instruments.

The Ethical Committee of Faculty of Medicine and Health Sciences, UPM has approved the study. Association between variables were examined by using Chi square and Fisher exact tests. Setting Confidence interval at 95% for mean score estimation, all significant levels were defined at standard *P* value of <0.05.

Data analyzing showed the prevalence of smoking, bullying, and being bulled among the study participants was 15.9%, 8.5%, and 19.0% respectively. Gender (being male) and parents’ marital status (living with a single parent) were identified as the risk factors of both smoking and bullying, while no significant relationship has found between the 2 risk behaviors and ethnicity, family income and the type of family. Moreover, 17.3% and 20.7% of the respondents reported to have insecurely attached to their mothers and their fathers respectively (Table 1).

**Table 1:** The relationship between attachment styles and smoking

***Attachment *** ***Styles***	***Smoking***		***Total n (%)***	***x^2^***	***df***	***P value***
	***Yes n (%)***	***No n (%)***				
Mother						
Insecure	81 (24.9)	244 (75.1)	325 (100)			
Secure	195 (14.2)	1183 (85.8)	1378 (100)	22.47	1	<0.001^*^
Father						
Insecure	92 (25.0)	276 (75.0)	368 (100)			
Secure	185 (14.1)	1128 (85.9)	1313 (100)	24.86	1	<0.001^*^

* Significant at *P* < 0.05

Insecure attachment style to mother (χ^2^=22.47, *P*<0.001) and father (χ^2^=24.86, *P*<0.001) had a positive significant relationship with smoking. As well, there was a significant association between bullying and insecure attachment styles to both mother (χ^2^=17.24, *P*<0.001) and father (χ^2^=19.63, *P*<0.001) (Table 2).

**Table 2:** The relationship between attachment styles and bulling

***Attachment *** ***Styles***	***Bullying***			***Total n (%)***	***x^2^***	***df***	***P value***
	***Bullying n (%)***	***Been bullied n (%)***	***Never Involved n (%)***				
Mother							
Insecure	38 (12.6)	73 (24.3)	190 (63.1)	301 (100)	17.24	2	
Secure	110 (7.6)	257 (17.8)	1075 (74.5)	1442 (100)			<0.001^*^
Father							
Insecure	41 (11.5)	90 (25.1)	227 (63.4)	358 (100)			
Secure	105(7.7)	234 (17.2)	1024 (75.1)	1363 (100)	19.63	2	<0.001^*^

* Significant at *P* < 0.05

In conclusion, a strong association was found between insecure attachment (both mother and father) with smoking and bullying risk behaviors among the secondary school students participating in this study. The study also has represented significant information about a potential factor which could contribute to smoking and bullying among adolescents at this locality. Therefore, the results of this study would be able to use by authorities and researchers concerning to initiate an intervention plans to reduce these risk behaviors. Future studies also may focus on the association strength of attachment styles with smoking and bullying compared to the other risk factors. As well, identifying individuals with insecure attachment style in early adolescence stage may help to decrease the prevalence of smoking and bullying among Malaysian youth.
